# Designing 3D Ternary Hybrid Composites Composed of Graphene, Biochar and Manganese Dioxide as High-Performance Supercapacitor Electrodes

**DOI:** 10.3390/nano13121866

**Published:** 2023-06-15

**Authors:** Vahid Babaahmadi, S. E. M. Pourhosseini, Omid Norouzi, Hamid Reza Naderi

**Affiliations:** 1 Materials and Textile Engineering Department, Faculty of Engineering, Razi University, Kermanshah 6714414971, Iran; 2 Faculty of Chemistry, University of Tehran, Tehran 1417935840, Iran; 3 Mechanical Engineering Program, School of Engineering, University of Guelph, Guelph, ON 1G 2W1, Canada

**Keywords:** activated carbon, graphene, supercapacitor, luffa, nanocomposite

## Abstract

Biochar derived from waste biomass has proven to be an encouraging novel electrode material in supercapacitors. In this work, luffa sponge-derived activated carbon with a special structure is produced through carbonization and KOH activation. The reduced graphene oxide (rGO) and manganese dioxide (MnO_2_) are in-situ synthesized on luffa-activated carbon (LAC) to improve the supercapacitive behavior. The structure and morphology of LAC, LAC-rGO and LAC-rGO-MnO_2_ are characterized by the employment of X-ray photoelectron spectroscopy (XPS), X-ray diffraction (XRD), BET analysis, Raman spectroscopy and scanning electron microscopy (SEM). The electrochemical performance of electrodes is performed in two and three-electrode systems. In the asymmetrical two-electrode system, the LAC-rGO-MnO_2_//Co_3_O_4_-rGO device shows high specific capacitance (SC), high-rate capability and excellent cycle reversibly in a wide potential window of 0–1.8 V. The maximum specific capacitance (SC) of the asymmetric device is 586 F g^−1^ at a scan rate of 2 mV s^−1^. More importantly, the LAC-rGO-MnO_2_//Co_3_O_4_-rGO device exhibits a specific energy of 31.4 W h kg^−1^ at a specific power of 400 W kg^−1^. Overall, the synergistic effect between the ternary structures of microporous LAC, rGO sheets and MnO_2_ nanoparticles leads to the introduction of high-performance hierarchical supercapacitor electrodes.

## 1. Introduction

A supercapacitor is a great supplement for energy conversion and high-rate power supply devices that deliver significant energy with high power density [1,2]. Recently, more interests have been focused on designing novel electronic devices by developing high-performance supercapacitors [3,4,5,6]. Due to their unique features, including high power density, fast charge and discharge rate, and long cycle life, supercapacitors are a promising substitute for current energy-storing devices [7,8]. Generally, Supercapacitors are divided into two categories: pseudocapacitors and electric double-layer capacitors (EDLCs) based on the charge storage mechanism [9]. A variety of materials have been chosen as possible electrodes in supercapacitors, including (1) carbonaceous structures (activated carbon, carbon nanotubes, carbon nanofiber, graphene) [10,11], (2) conductive polymers (polypyrrole, polyaniline) [12,13] and (3) transition metal oxides (RuO_2_, MnO_2_, V_2_O_5_, Fe_2_O_3_, SnO_2_) [14]. The combination mechanisms (faradaic and non-faradaic) as hybrid supercapacitors (HSCs) have been prepared by using composite electrodes or cell configuration (asymmetric) to achieve higher performances [15,16]. The agricultural precursors are low price, environmentally friendly, more available, and porosity-intrinsic [17], include clover stems [18], lotus leaves [19], pueraria [20], rice husk [21], bamboo [22], coconut shell [23], dead leaves [24], hemp [25], cattail [26], sugarcane bagasse [27], sunflower seed shells [28], corn husk [29] and luffa sponge [15,30,31,32,33,34] which have been successfully converted into ACs. These materials could be further processed by chemical, physical, and a combination of carbonization and activation processes to reach a desired surface topography and morphology [26,35]. For example, hydrothermal carbonization in the presence of KOH has been considered an efficient technique to convert precursors into a superior three-dimensional (3D) morphology and improve the surface area and functional groups [36]. To date, several studies have attempted to modify luffa and obtain micrometer-scale channels [30,32], three-dimensional and hierarchical porous [31,34], carbon quantum dots (CQDs) [33], carbon/metal oxide porous [15] and activated carbon structures. Different composite materials made from carbonaceous structures containing MnO_2_ pseudocapacitors have been studied and presented as novel active materials in HSC electrodes due to their high capacitance, higher energy density and long cycle life [37,38]. The narrow size distribution of micropores in graphene provides a high surface area that would favor the EDLC performance, while activated carbon, derived from organic wastes, can create a 3D interconnected structure [30,31,32]. Scientific literature and studies on the development of metal oxide-carbon material electrodes utilizing high capacitance of metal oxide (pseudocapacitance) and activated carbon (EDLC) along with high electrical conductivity of graphene [37,38,39]. In the present work, a novel ternary composite structure has been designed based on luffa-activated carbon-reduced graphene oxide-metal oxide (LAC-rGO-MnO_2_) to fabricate high-performance asymmetric supercapacitors. The LAC works as a good 3D substrate for ion transport and capacitor devices based on a uniquely dense and intrinsic multi-channeled arrangement structure [40]. The LAC-rGO is expected to be a great substrate for accommodating MnO_2_ nanoparticles based on unique physiochemical properties. The hierarchical 3D structure of LAC, in-situ chemical reduction and metal oxide decoration were obtained by a facile procedure involving the simultaneous hydrothermal carbonization and chemical activation of bio-based precursor and GO reduction. The main objectives of this work were to (i) simultaneously transform luffa sponge into AC along with the in-situ hydrothermal reduction of GO sheets followed by further MnO_2_ decoration, (ii) to discuss the electrochemical properties of the different composite electrodes, and (iii) to assemble a high-performance asymmetric supercapacitor made of ternary composite electrodes.

## 2. Materials and Methods

**Figure 1:**
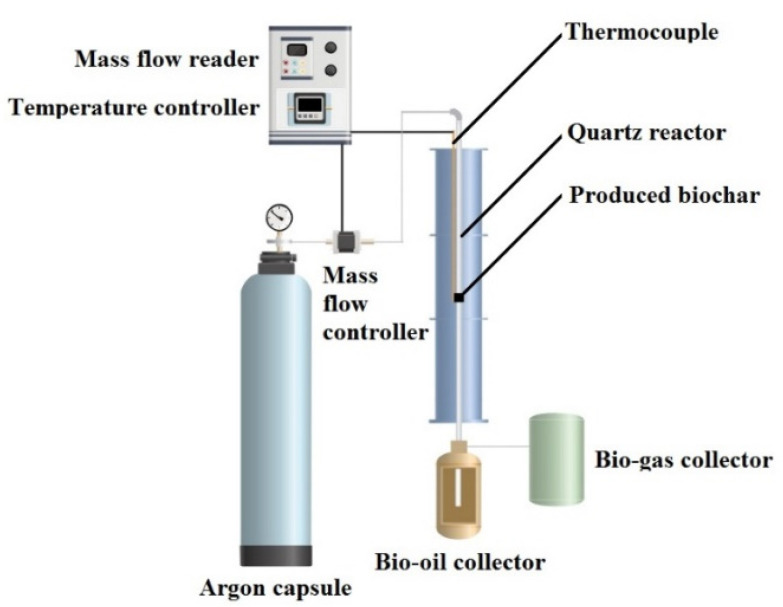
Schematic of carbonization and activation process of luffa sponge to produce biochar under the argon atmosphere (30 mL min^−1^ flow rate) at temperatures in the range of 30–800 °C (heating rate, 10 °C min^−1^).

Potassium hydroxide, sodium sulfate, sulfuric acid (98%), hydrochloric acid (30%) and hydrogen peroxide (30%) were used in analytical grade from Merck (Rahway, NJ, USA) without any further purifications. Graphite powder (natural graphite flakes, Asbury graphite mills, Inc., Asbury, NJ, USA) and Luffa sponge was purchased from a local market and washed to remove the impurities. The whole conditions for preparing the nanocomposites and carbonization processes were controlled under the argon atmosphere, which is shown in Figure 1.

### 2.1. Preparation of Luffa Carbon (LC) and Luffa Activated Carbon (LAC)

For preparing LAC, 2 g of dried and ball-milled luffa sponge was added to potassium hydroxide solution (6 M) and refluxed for 6 h at 100 °C. This mixture was centrifuged and then pyrolyzed at 800 °C for 2 h. To remove the additional potassium hydroxide, the as-produced biochar was washed with 1 M HCl and followed by rinsing with pour water and finally dried at 120 °C overnight. The LC was prepared with the same condition without any KOH activation.

### 2.2. Preparation of LAC-rGO Nanocomposite

The graphene oxide powder was produced by modified Hummer’s oxidation route. Typically, a 10 mL GO solution (5.0 mg mL^−1^) and 2 g dried Luffa powder were placed in an 80 mL Teflon-lined autoclave and maintained for several hours to expand the luffa and heated at 180 °C for 12 h. The prepared material after hydrothermal products were refluxed in the presence of 6 M KOH for about 6 h at 100 °C. Then, this mixture was centrifuged and pyrolyzed at 800 °C.

### 2.3. Preparation of LAC-rGO-MnO_2_ Nanocomposite

The LAC-rGO (50 mg) was added to KMnO4 aqueous solution (1.5 g L^−1^) while stirring. Then, the pH of the precursor solution was adjusted to 4 by adding 0.5 M HCl. Subsequently, the mixture was stirred for 2 h at 60 °C to obtain LAC-rGO-MnO_2_ nanocomposite.

### 2.4. Structural Characterization

Different characterization methods have been used to clarify the physical properties, chemical structure and morphology of the final nanocomposites. The FESEM (MIRA3 LM, Tescan, Brno, Czech Republic), Raman spectroscopy (Takram P50C0R10, Tehran, Iran), XRD (Xpert MPD, Malvern, UK), and XPS (PerkinElmer PHI 6000C ESCA, Waltham, MA, USA) (system with monochromic Al KR (1486.6 eV) irradiation), were performed to determine the properties of the nanocomposite. The d-spacing (d_002_) of graphitic layers was calculated based on Bragg’s law from XRD data. Thermogravimetric analysis (TGA) was carried out on a thermoanalyzer (Pyris Diamond TG/DTA, Waltham, MA, USA). The specific surface area and pore size distribution were obtained using Bruauere-Emmette-Teller (BET) and Barrette-Joynere-Halenda (BJH) methods, respectively, based on the adsorption and desorption behavior of the N_2_.

### 2.5. Electrochemical Measurements 

Electrochemical measurements for supercapacitive studies (EIS, CV and GCD measurements) were carried out by using a potentiostat/galvanostat workstation (PGSTAT302N, Autolab, Ultrecht, The Netherland) in 0.5 M Na_2_SO_4_ aqueous solution. Electrochemical tests were performed on two and three-electrode systems. The working electrodes were fabricated for the electrochemical measurements by a mixture of the synthesized samples (LAC, LAC-rGO, LAC-rGO-MnO_2_) with carbon black, graphite and polytetrafluoroethylene at a 65:10:20:5 mass ratio, then dispersed in ethanol. In order to distribute suspension over a current collector, a piece of rustproof steel, about 5 mg of the electro-active material and a 1 cm^2^ current collector were utilized for this purpose. Finally, the electrodes were dried in a vacuum oven at 80 °C for 1 h. Ag/AgCl as reference and platinum as counter electrodes were applied in electrochemical measurement. An asymmetric cell was assembled by using the LAC-rGO-MnO_2_ as a positive electrode which is produced in this work, and the Co_3_O_4_/rGO as a negative electrode in our previous works [41]. In this research, the SC is calculated from the integration of CV curves. For the three-electrode system, the potentiostatic SC (F g^−1^) was calculated by this equation:

(1) $$\mathrm{SC}=\frac{\int_{\mathrm{Va}}^{\mathrm{Vc}}I\left(V\right)\mathrm{dV}}{2\times m\times v\times \Delta V}$$

I is the current density of the CV, ν is the scan rate, m is the mass of the electroactive material, and ΔV (Vc-Va) is the potential window.

For the two-electrode system, the SC for a single electrode was calculated by this equation:

(2) $$\mathrm{SC}=\frac{2\int_{\mathrm{Va}}^{\mathrm{Vc}}I\left(V\right)\mathrm{dV}}{m\times v\times \Delta V}$$

## 3. Results and Discussion

**Figure 2:**
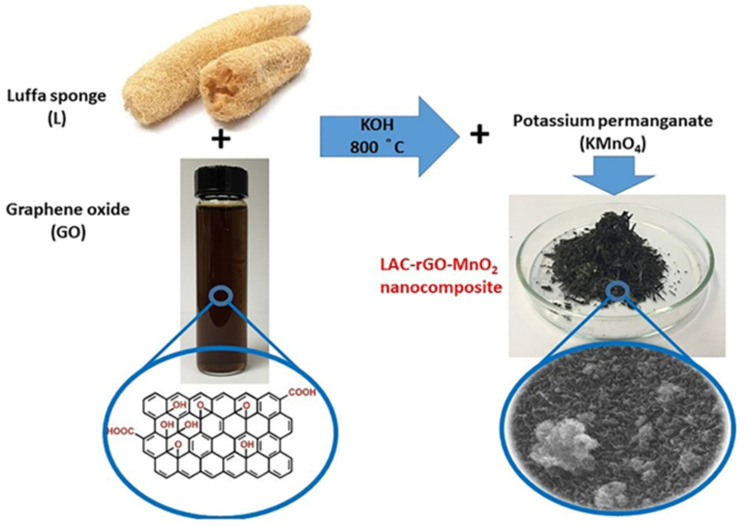
Schematic view of the carbonization-activation along with in-situ chemical reduction to prepare LAC-rGO-MnO_2_ nanocomposite.

A simultaneous/consecutive process has been developed to prepare an activated carbon-graphene-metal oxide nanocomposite structure as a high-performance supercapacitor electrode. In this process, the bio-based luffa sponge has been properly carbonized and activated along with in-situ thermo-chemical reduction of GO, followed by the deposition of MnO_2_ nanoparticles. To better explain the material, the final product (LAC-rGO-MnO_2_) is like a tree with LAC and rGO as branches and MnO_2_ as a leaf to produce a fast and reversible ion transfer network. Figure 2 shows the whole process regarding carbonization and in-situ loading of rGO-MnO_2_ on activated carbon structure. 

### 3.1. Characterization of Nanocomposite Electrodes

**Figure 3:**
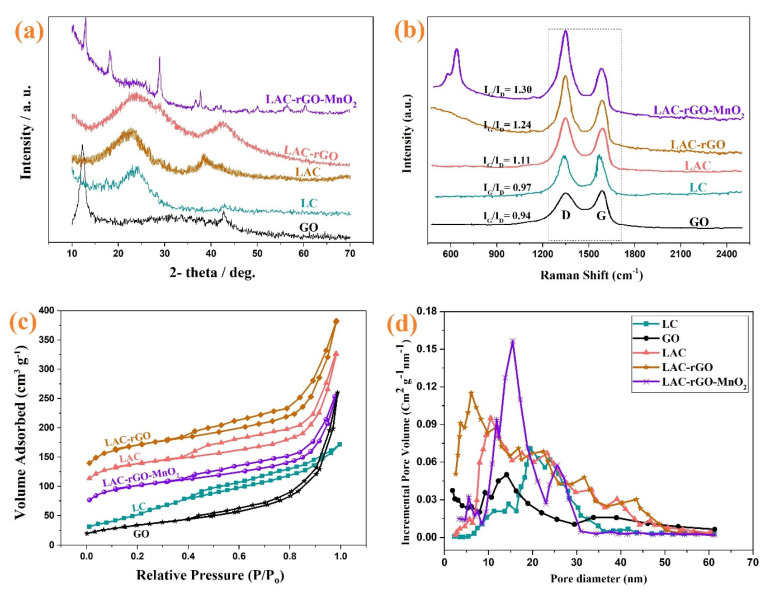
XRD spectra of GO, LC, LAC, LAC-rGO and LAC-rGO-MnO_2_ nanocomposites (**a**), Raman spectra of GO, LC, LAC, LAC-rGO, LAC-rGO-MnO_2_ nanocomposites (**b**), N2 adsorption and desorption isotherms (**c**), and BJH pore size distribution of GO, LC, LAC, LAC-rGO, and LAC-rGO-MnO_2_ nanocomposites (**d**).

Figure 3 shows the XRD, Raman spectroscopy and BET characterization of GO, LC, LAC, LAC-rGO and LAC-rGO-MnO_2_ samples. Figure 3a exhibits the XRD spectra of the different samples, which are measured in the range of 10° to 70°. The pattern of synthesized GO powder shows an obvious diffraction peak (001) at 2θ = 12.3° corresponding to the distance between the basal plane of graphitic layers (of about 0.72 nm) and a small diffraction peak (100) at 2θ = 42.8° due to graphitization [42]. The XRD spectra of LC and LAC show two obvious peaks locate around 23–25° and 42–45°, corresponding to the diffraction of (002) and (100) planes of the graphitic structure [32]. The (002) peak shifted to the lower degree in LAC and yielded a broad peak (22°) which is believed to originate from the functionalities of carbon lattice. A peak shift in LAC-rGO comes from the decomposition of oxygenated functional groups in GO sheets through the in-situ reduction and activation process. KOH and carbonization processes work as a co-reduction condition which would transform GO into rGO along with activation of the carbon structure. The characteristic peaks of LAC-rGO-MnO_2_ composite at 12.7°, 18°, 28°, 37.5°, 42.1°, 49.9°, 56° and 61° can be indexed to α-MnO_2_ (JCPDS 44-0141) corresponded to (110), (200), (310), (211), (301), (411), (600) and (521) crystal planes indicating successful loading of MnO_2_ on carbon substrate which is in agreement with and further support the results of FESEM images [43]. The broad peak of the graphitic layer in LAC-rGO and LAC-rGO-MnO_2_ is related to the structure of exfoliated rGO layers. Furthermore, the peak disappeared (100) after the decoration of MnO_2_, resulting based on the erosion of carbons in the GO structure after a redox reaction [1].

Raman spectroscopy is a non-destructive technique that can also be used to analyze carbonaceous materials. The carbonaceous structure shows D (~1345 cm^−1^) and G (~1585 cm^−1^) bands caused by the disordered structure and E_2g_ mode of the sp2 lattice, respectively. The ratio of two peaks (I_D_/I_G_) indicates the degree of graphitization of the material. This ratio (ID/IG) is much higher for LAC-rGO-MnO_2_ and LAC-rGO than for LC, which indicates that the carbon structure is graphitized during the activation and compositing process (Figure 3b). In the spectrum for LAC-rGO-MnO_2_, a peak is observed at 601 cm^−1^, which is related to the symmetric stretching vibration of the Mn-O bond in MnO_2_ [44]. The BET method is employed to characterize the surface area of highly porous materials, as shown in Figure 3c,d. The shape of isotherms belongs to I/IV (microporous and mesoporous materials) with a hysteresis loop of type H4 based on IUPAC classification and data [45]. The mesopores structure provides the diffusion channel for the fast movement of electrolyte ions in charge and discharge times [46]. According to the N_2_ adsorption isotherms, BET surface areas of GO, LC, LAC, LAC-rGO and LAC-rGO-MnO_2_ were calculated to be 119, 179, 484, 595, 356 m^2^ g^−1^, respectively. The N_2_ adsorption increases through chemical activation and nanostructure loading, which indicates the development of the high-surface mesoporous structure. Further, in the presence of rGO, the surface area increases up to 595 m^2^ g^−1^ with a wide hysteresis loop, confirming that porosity was developed largely during simultaneous in-situ chemical activation and reduction (Table 1). It also may come based on thermal deoxygenation and produce different gases in reduction, which creates more vacancies.

**Figure 4:**
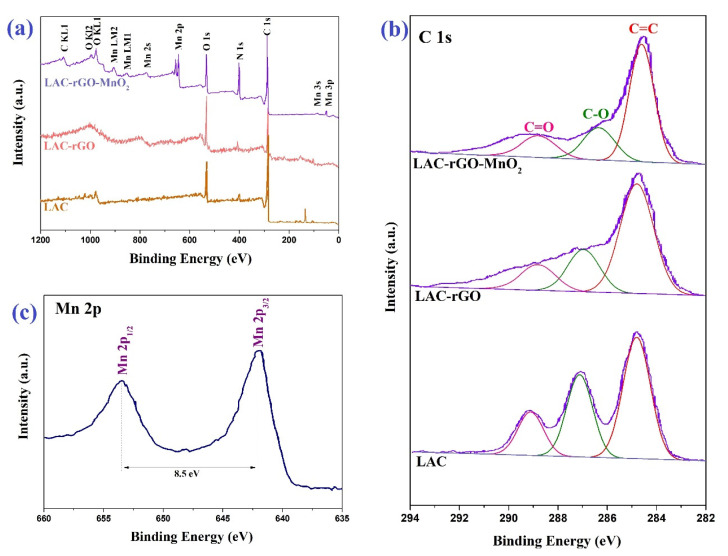
XPS spectra of LAC, LAC-rGO and LAC-rGO-MnO_2_ (**a**), high-resolution of C1s of LAC, LAC-rGO and LAC-rGO-MnO_2_ (**b**) and high-resolution of Mn 2p of LAC-rGO-MnO_2_ (**c**).

In the XPS spectra of the survey scan, high-resolution C1s and Mn 2p orbitals of the LAC, LAC-rGO and LAC-rGO-MnO_2_ composites are shown in Figure 4. The high-resolution spectra of C1s can be deconvoluted as three peaks at 284.7, 286.5, and 288.8 eV, implying C=C, C–O and C=O bonds (Figure 4b). As shown in Figure 4c, the peaks of Mn 2p_3/2_ and Mn 2p_1/2_ are centered at 641.0 and 653.5 eV, respectively, with energy separation of 11.5 eV, in good match with pure MnO_2_ in crystal lattice [38]. Based on Elemental composition, the atomic percentages of C, O and Mn are determined to be 39.6, 26.5, and 31.3 %, respectively.

**Figure 5:**
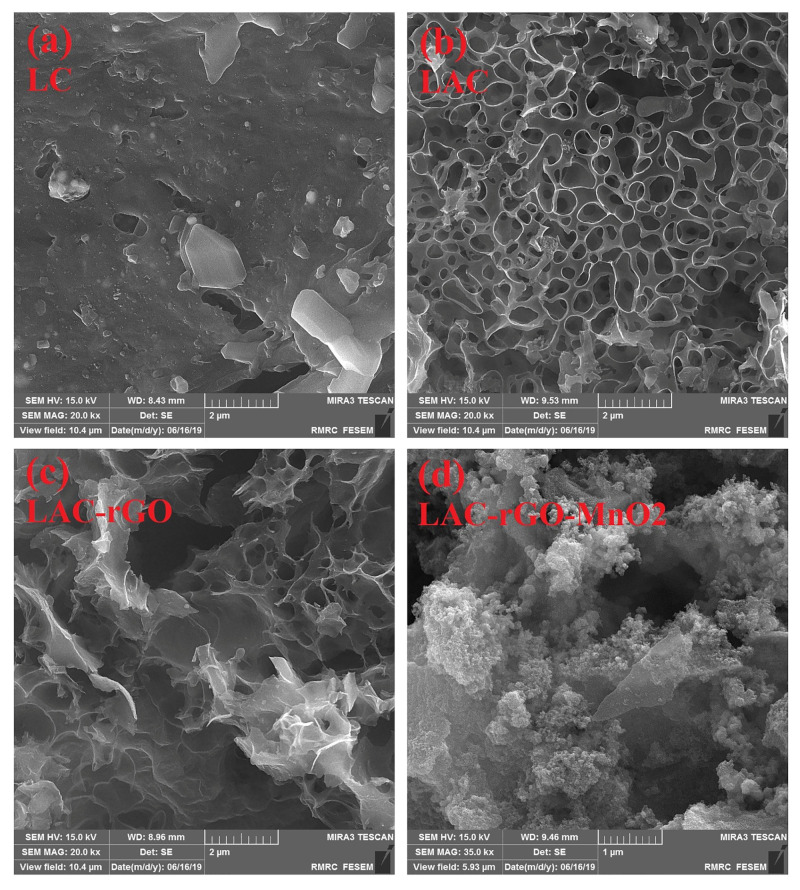
FESEM images of LC (**a**), LAC (**b**), LAC-rGO (**c**), LAC-rGO-MnO_2_ (**d**).

**Figure 6:**
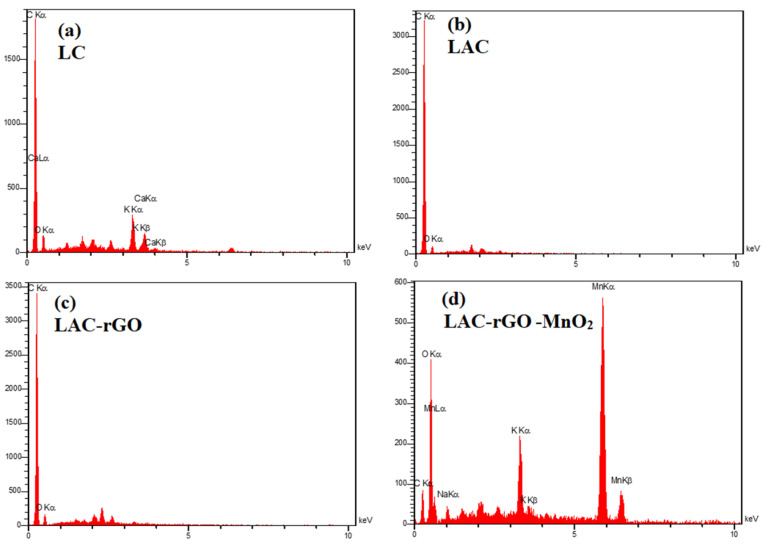
EDS analysis of LC (**a**), LAC (**b**), LAC-rGO (**c**), LAC-rGO-MnO_2_ (**d**).

The surface morphology of the LA and LAC structures in the presence of rGO and MnO_2_ nanostructures have been investigated by FESEM (Figure 5). Figure 5a,b illustrates that the activated sample (LAC) has higher porous morphology and open sites in comparison to LC. The in-situ reduction of GO, along with carbonization and activation (Figure 5c), tends to produce a hierarchical high surface area structure which is confirmed by BET results (Table 1). The loading of MnO_2_ nanoparticles on the LAC-rGO surface is clear in Figure 5d as nanocomposite decorations to obtain multifunctional activity in electrochemical performance. The EDS analysis of all the samples is shown in Figure 6. 

The KOH activation of carbon structures increases the specific surface area, uniform pore size and controls the functional groups on the surface based on condition and detail. The side products of KOH activation and in-situ reduction were potassium oxide (K_2_O), potassium carbonate (K_2_CO_3_), H_2_, H_2_O, CO and CO_2_, which help to boost the porosity [47]. All the processes mentioned combine simultaneous and consecutive reactions, (1) carbonization of luffa, (2) KOH activation of luffa carbon, (3) KOH reduction of GO, and (4) in-situ thermal reduction of GO within carbonaceous substrate reveal gas evolution based on the decomposition of oxygen-containing groups. The mentioned gases have positive effects on porosity through the pressure induced by the exhaustion from the inner layer up to the outer surface and the remaining vacancies. 

### 3.2. Electrochemical Characterization

**Figure 7:**
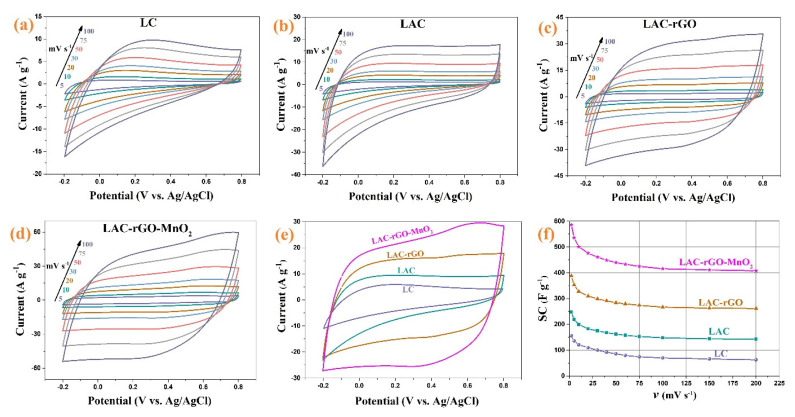
The CV of LC, LAC, LAC-rGO and LAC-rGO-MnO_2_ electrodes at different sweep rates (**a**–**d**), the CV curves of LC, LAC, LAC-rGO and LAC-rGO-MnO_2_ electrodes at 50 mV s^−1^ (**e**), the SC vs scan rates for LC, LAC, LAC-rGO and LAC-rGO-MnO_2_ electrodes (**f**).

The electrochemical performance was conducted in a three and two-electrode cell in an aqueous solution for electrodes and asymmetric device structure. Figure 7a–d shows the CV curves of LC, LAC, LAC-rGO and LAC-rGO-MnO_2_ composite electrodes at various scan rates between −0.2 and +0.8 V. Figure 7e shows the compared CV curves of prepared electrodes at scan rate 50 mV s^−1^. As shown in the presence of rGO and MnO_2_, the CV curves are rectangular in shape and reveal a mirror image, which means ideal capacitive behavior on the potential window. Figure 7f shows the SC variation trend of different composite electrodes at various sweep rates (2 to 200 mV s^−1^). The SC of the different electrodes was calculated at 156, 248, 388, and 586 F g^−1^ for LC, LAC, LAC-rGO and LAC-rGO-MnO_2_ electrodes, respectively, at a sweep rate of 2 mVs^−1^. The LAC exhibits higher SC compared to LC in all scan rates. The LAC-rGO-MnO_2_ composite exhibits the highest SC values and remains at 70% at a high rate compared to all other samples. The interfacial of MnO_2_ and LAC-rGO is a key point to the fast transfer of electrons into the electrode structure. Thus, the excellent specific capacitance of the LAC-rGO-MnO_2_ electrode corresponded to the high microporous activated surface area (BET data), increased electrical conductivity and both electric double layer and pseudocapacitance behavior.

**Figure 8:**
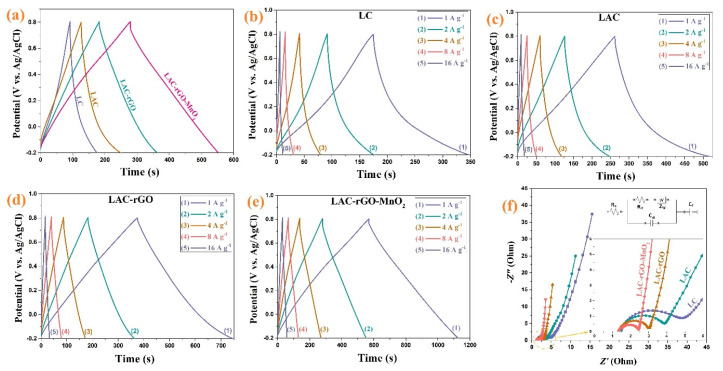
The GCD curves of the LC, LAC, LAC-rGO and LAC-rGO-MnO_2_ electrodes at constant current density of 2 A g^−1^ (**a**), The GCD curves of the LC, LAC, LAC-rGO and LAC-rGO-MnO_2_ electrodes at various current densities (1, 2, 4, 8, 16 A g^−1^) (**b**–**e**), and Nyquist plots of LC, LAC, LAC-rGO and LAC-rGO-MnO_2_ electrodes (**f**).

Figure 8a compares the charge-discharge behavior of different samples between −0.2 and +0.8 V at constant current density 2 A g^−1^. The nearly symmetric and linear shapes of charge–discharge curves show a fast and reversible reaction on the surface of electrodes, as was shown previously in CV curves as an excellent capacitive behavior. Figure 8b–e shows the charge-discharge curves of the LC, LAC, LAC-rGO and LAC-rGO-MnO_2_ electrodes at various current densities (1–16 A g^−1^). The galvanostatic charge-discharge curves of LAC-rGO-MnO_2_ electrodes in various current densities are triangular-shaped, linear, symmetric, and sharp. Furthermore, the reversible behavior, high columbic efficiency, and the ideal capacitor performance of the LAC-rGO-MnO_2_ electrode can be concluded from the equal durations of charging and discharging. This is due to the uniform distribution of MnO_2_ nanoparticles on the LAC-rGO structure, resulting in improved electrical conductivity and fast redox reactions. In addition, the LAC-rGO-MnO_2_ electrode shows a lower IR drop in the same current density compared with the LC, LAC and LAC-rGO electrodes. These results reveal the existence of higher accessible electroactive sites in the LAC-rGO-MnO_2_ electrode compared to other electrodes.

For further understanding, the EIS test was conducted at open circuit potential in the frequency range of 100 kHz–0.01 Hz (Figure 8f). It is revealed that LC, LAC, LAC-rGO and LAC-rGO-MnO_2_ electrodes show a vertical line in the low-frequency region and almost the same shape with a loop at a high-frequency region based on ideally combined capacitive behavior. As shown in the high-frequency region, all the EIS curves intercept the real axis (inset in Figure 8f). The EIS spectra are fitted by the electrical equivalent circuit proposed in Figure 8f (inset). The complex nonlinear least squares (CNLS) fitting method is commonly used to fit and simulate impedance data. With the activation of carbon, the electrolyte resistance (Rs) and charge transfer resistance (Rct) was reduced (Table 2). Furthermore, with the addition of rGO and MnO_2_, this reduction in Rs and Rct has become more dramatic. Another important parameter in a fitted circuit is the faradic capacitance (CF), which indicates the amount of redox reaction that takes place at the electrode surface. As expected, the CF increases dramatically with the addition of MnO_2_ to the carbon structure (LAC-rGO). In general, the parameters fitted with the electrical circuit show the excellent supercapacitor performance of the composite electrode) LAC-rGO-MnO_2_ (compared to other electrodes.

**Figure 9:**
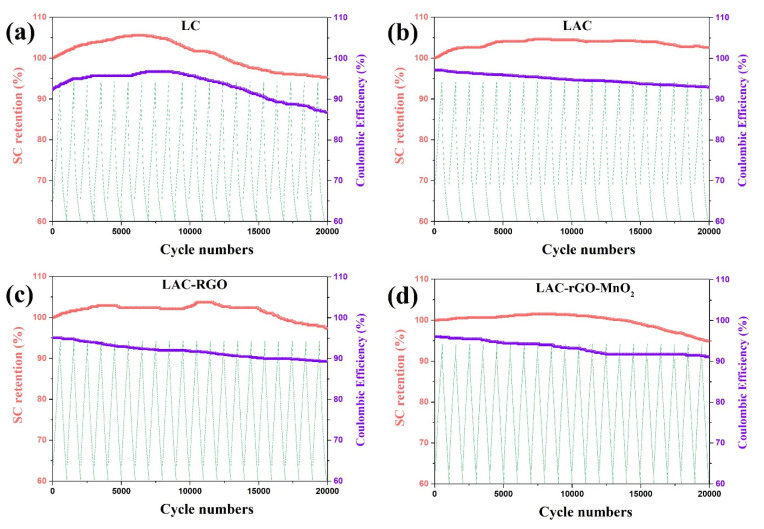
Cyclic performance of the LC (**a**), LAC (**b**), LAC-rGO (**c**) and LAC-rGO-MnO_2_ (**d**) electrodes at a current density of 16 A g^−1^.

The cycling life of over 20,000 continuous charge/discharge for all electrodes was tested at a current density of 16 A g^−1^. Figure 9 illustrates the retention of SC as a function of the charge/discharge cycles for all sample electrodes. The composite electrodes exhibit excellent electrochemical stability without any declination of the initial SC over 20,000 cycles. The increase of capacitance retention occurs based on better wetting and swelling of the micro-scale channels electrode with activation of new sites in the electrodes, which increases the charge transferability [41]. Increasing the number of electroactive sites in electrodes with the extended charge–discharge cycles is principally responsible for the rapid reactions, and hence the cycling stability increases. Moreover, the electrodes exhibit a superior coulombic efficiency after 20,000 cycles [48]. Table 3 shows the comparison of supercapacitive behavior between LAC-rGO-MnO_2_ and reported luffa-derived activated carbon composites.

**Figure 10:**
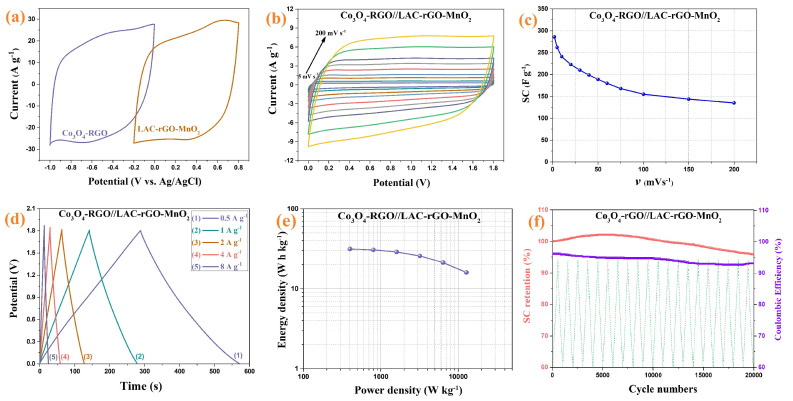
Comparison CV of Co_3_O_4_/rGO and LAC-rGO-MnO_2_ electrodes at a scan rate of 50 mV s^−1^ (**a**), CV curves of Co_3_O_4_/rGO//LAC-rGO-MnO_2_ HSC at various scan rates (**b**), The SC variation of Co_3_O_4_/rGO//LAC-rGO-MnO_2_ HSC with scan rate (**c**), charge/discharge curves at various current densities (**d**), The Ragone plots of Co_3_O_4_/rGO//LAC-rGO-MnO_2_ HSCs € and Cycle performance of Co_3_O_4_/rGO//LAC-rGO-MnO_2_ HSC after 20,000 charge-discharge (**f**).

Based on the electrochemical measurement, the LAC-rGO-MnO_2_ was selected as the best composite electrode material in this research to assemble an asymmetric hybrid supercapacitor. The Co_3_O_4_/rGO composite electrode with a well-known characterized structure and electrochemical properties—which was published in previous research [41]—has been used as a negative electrode. The overall electrochemical measurement of assembled Co_3_O_4_/rGO//LAC-rGO-MnO_2_ HSC is shown in Figure 10. It was founded that the positive and negative electrodes possess a potential window from −0.2 to 0.8 V and −1 to 0 V, respectively (Figure 10a). Thus, for the obtained asymmetric supercapacitor, the final cell voltage which can be used is the sum of the potential window of two electrodes. Figure 10b shows the optimized CV curves of Co_3_O_4_/rGO//LAC-rGO-MnO_2_ HSC at various scan rates (5 to 200 mV s^−1^) with a potential window of 1.8 V. The fabricated asymmetric supercapacitor shows an ideal capacitive behavior with a nearly rectangular shape in CV curves with a potential window of 1.8 V. The SC variation trends as a function of scan rate are shown in Figure 10c. As shown, at the higher scan rates, the SC decreases because the time constraint limits the diffusion and movement of ions [1]. At the scan rate of 2 mV s^−1^, the maximum amounts of SC for Co_3_O_4_/rGO//LAC-rGO-MnO_2_ HSC were obtained 286 F g^−1^. The decrease of a maximum of 52.8% in the SC, even at scan rates as high as 200 mV s^−1^, is an indication of the outstanding capacitive retaining tendency of the HSC device. 

Figure 10d shows the GCD curves of the Co_3_O_4_/rGO//LAC-rGO-MnO_2_ HSC at different current densities. The GCD curves of Co_3_O_4_/rGO//LAC-rGO-MnO_2_ HSC were triangular and symmetric. In addition, these curves revealed a fast I–V response, low ESR, and ideal capacitive behavior, which was also confirmed by CV curves. The specific power and specific energy of the Co_3_O_4_/rGO//LAC-rGO-MnO_2_ HSC as Ragone plots are shown in Figure 10e. The present high performance of the Co_3_O_4_/rGO//LAC-rGO-MnO_2_ HSC device exhibits excellent specific energy (31.4 W h kg^−1^ at 400 W kg^−1^). Compared with the other reported HSC devices, including functional biochar//Fe composite biochar (30.8 W h kg^−1^ at 1000 W kg^−1^) [16], graphene hydrogel//MnO_2_ on nickel foam (23.2 W h kg^−1^ at 1000 W kg^−1^) [50], MnO_2_//graphene (25.2 W h kg^−1^ at 2100 W kg^−1^) [51], MnO_2_ nanotubes//Active carbon-carbon nanotubes (24.7 W h kg^−1^ at 100 W kg^−1^) [52], MnO_2_-Graphitic Carbon Spheres//Graphitic Carbon Spheres (22.1 W h kg^−1^ at 7000 W kg^−1^) [53], and graphene-MnO_2_//graphene (10.0 W h kg^−1^ at 2530 W kg^−1^) [54], Co_3_O_4_/rGO//LAC-rGO-MnO_2_ HSC device illustrated more supercapacitive performances with a high specific energy.

The cycling stability of electrodes is an important parameter for supercapacitor devices. The stability study of Co_3_O_4_/rGO//LAC-rGO-MnO_2_ HSC was performed at a current density of 8 A g^−1^ for 20,000 continuous charge/discharge (Figure 10f). The Co_3_O_4_/rGO//LAC-rGO-MnO_2_ HSC device shows excellent stability during the 20,000 cycles (96.1%). The electrochemical performance of Co_3_O_4_/rGO//LAC-rGO-MnO_2_ HSC shows a supercapacitor device with excellent properties and good rate capability.

## 4. Conclusions

This study showed that luffa sponge biochar was utilized as a high-performance electrode for supercapacitors. The luffa sponge was first activated with KOH solution and then combined with rGO and MnO_2_ to further increase the SC and improve the electrochemical behavior. The LAC shows a nearly ideal supercapacitive behavior with proper SC and cycle stability. The increase of SC in the LAC-rGO-MnO_2_ composite electrode was related to the redox reaction of MnO_2_ and the high conductivity of rGO sheets. This composite electrode shows the appropriate specific capacitance (586 F g^−1^) and high cycle stability (95% after 20,000 CV cycles). The synergistic behavior of the material in composites has been verified via CV and EIS data, demonstrating a larger area of the CV curve and lower Rct (1.1 Ω) of the LAC-rGO-MnO_2_ electrode. The asymmetric supercapacitor of LAC-rGO-MnO_2_//rGO-Co_3_O_4_ shows good capacitive behavior with an excellent SC of 286 F g^−1^ at 2 mV s^−1^ and a great cycle stability of 96.1% after 20,000 charge/discharge cycles. In conclusion, this research shows a good way to obtain new materials with excellent energy storage properties.

## Figures and Tables

**Table 1:** BET and BJH analysis data for GO, LC, LAC, LAC-rGO and LAC-rGO-MnO_2_.

Sample	GO	LC	LAC	LAC-rGO	LAC-rGO-MnO_2_
Surface Area (m^2^ g^−1^)	119	179	484	595	356
Pore Volume (cm^3^ g^−1^)	0.113	0.198	0.414	0.523	0.338
Pore Size (nm)	14.1	19.5	10.5	6.1	15.5

**Table 2:** The fitted values with equivalent circuit of the experimental impedance spectra.

Values	LC	LAC	LAC-rGO	LAC-rGO-MnO_2_
Rs	1.53	1.45	1.35	1.32
Rct	3.03	2.20	1.60	1.10
Q1 (mF)	0.38	0.33	0.45	0.21
n	0.92	0.93	0.90	0.84
W	0.13	0.15	0.41	0.69
CF (mF)	95	121	133	247

**Table 3:** Comparison of supercapacitive behavior of between LAC-rGO-MnO_2_ and reported luffa-derived activated carbon composite.

No.	Electrode Materials	Electrolyte	Scan Rate/Current Density	Specific CaPacitance (F g^−1^)	Capacitance Retention(%)	Ref.
1	Luffa-derived activated carbon MnO_2_ //N-doping porous carbon	1.0 M Na_2_SO_4_	1 A g^−1^	78.2	91.2 2000 cycles	[15]
2	Luffa-derived activated carbon	1.0 M Na_2_SO_4_	1 A g^−1^	167	95.7 5000 cycle	[30]
3	Luffa-derived activated carbon	6 M KOH	1 A g^−1^	304	98 10,000 cycles	[31]
4	Luffa-derived activated carbon	6 M KOH	1 A g^−1^	309.6	81.3 10,000 cycles	[34]
5	Luffa-derived activated carbon	6 M KOH	0.1 A g^−1^	82.3	-	[40]
6	Luffa-derived activated carbon	1 M NaCl	5 mV s^−1^	93.0	92.6 5000 cycles	[49]
7	Luffa-derived activated carbon-rGO-MnO_2_	0.5 M Na_2_SO_4_	2 mV s^−1^	586	95 20,000 cycles	This work

## Data Availability

Not applicable.
